# Predicting Successful Pulmonary Vein Isolation In Patients With Atrial Fibrillation By Brain Natriuretic Peptide Plasma Levels

**Published:** 2009-09-01

**Authors:** Dong-In Shin, Thomas Deneke, Eduard Gorr, Helge Anders, Kathrin Buenz, Marcus Paesler, Marc Horlitz

**Affiliations:** Department of Cardiology and Electrophysiology, Krankenhaus Porz am Rhein, Cologne, Germany

**Keywords:** Atrial fibrillation, BNP, ablation, pulmonary vein isolation

## Abstract

**Background:**

Catheter ablation for atrial fibrillation is a clinically established treatment by now while success rate varies between 60%  and 85%. Interventional treatment of atrial fibrillation is still a challenging technique associated with a long procedure time and risk of major complications in up to 6 % of treated patients. The aim of this study was to investigate the predictive value of plasma brain natriuretic peptide (BNP) in patients undergoing pulmonary vein isolation concerning stable sinus rhythm after ablation.

**Methods:**

In 68 consecutive patients with atrial fibrillation  (AF) and normal left ventricular ejection fraction, BNP was measured at baseline before pulmonary vein isolation (PVI). All patients received a 7-days-holter monitoring 3 months after radiofrequency (RF) ablation in order to detect recurrent AF episodes.

**Results:**

48 patients with paroxysmal and 20 patients with persistent AF were enrolled. Baseline BNP was significantly higher in patients with persistent AF compared to patients with paroxysmal AF (145,5 pg/ml vs. 84,4 pg/ml; p<0,05). 3 months after PVI 38 patients (79,1%) with paroxysmal AF had a stable sinus rhythm documented on 7-days-holter monitoring, where as in 10 patients (20,9%) AF episodes were detected. Patients with a successful PVI showed  significantly lower BNP plasma levels at baseline compared to patients with AF recurrrence (68,7 pg/ml vs. 144,1 pg/ml; p<0,05). In patients with persistent AF 55% (11 cases) had no recurrence of AF at 3 months 7-days holter and in 9 patients (45%) AF recurred. BNP plasma levels at baseline were lower in patients with stable sinusrhythm after 3 months compared to the group of recurrent AF (105,8 pg/ml vs. 193,3 pg/ml; p=0,11).

**Conclusions:**

Patients with AF and low preprocedural BNP plasma levels showed a better outcome after PVI. Thus BNP may be helpful in patient selection for a successful treatment of AF by PVI.

## Introduction

Pulmonary vein isolation (PVI) has been established as an effective tool in the treatment of drug refractory atrial fibrillation (AF). However PVI is still associated with long procedure duration and remains a challenging technique; furthermore extended left atrial ablation always contains the risk of major complication like stroke or pericardial tamponade. In consideration of these procedure specific aspects and a success rate of 60 to 85% [1,2], it seems to be crucial to assure an optimized patient selection. The purpose of this study was to investigate the predictive value of plasma brain natriuretic peptide (BNP) for a successfull PVI in patients with AF and normal left ventricular ejection fraction.

## Material and Methods

### Patient Characteristics

The study population was selected from 68 consecutive symptomatic AF patients - 53 men, (28 to 70 years), 48 with paroxysmal AF, and 20 with persistent AF. No patient had structural heart disease. All patients showed a normal left ventricular ejection fraction of greater than 55% applying echocardiographic criteria. Mean duration of atrial fibrillation was 3,8 years (8 months to 6 years). In 39 (58%) patients left atrial (LA) had a normal volume of <20cm^2^, in 18 (26%) patients LA was slightly enlarged (20 to 25 cm^2^), and in 11 (16%) patients LA volume was measured with to be≥25 cm^2^. All patients provided written informed consent and the study was approved by the institutional review board.

### Electrophysiological study and PVI procedure

Right femoral venous access was obtained and a multipolar electrode catheter was introduced into the distal coronary sinus. After transseptal catheterization a 4mm tip deflectable catheter (Navistar, Biosense Webster) was advanced into the left atrium and a 3-dimensional shell representing the left atrium was constructed by using an electroanatomical mapping system (CARTO, Biosense Webster). A CT image of the left atrium was integrated using a commercially available software (CARTOMERGE, Biosense Webster). Left atrial ablation was performed to encircle the left- and right sided pulmonary veins, with additional lines at the left atrial roof and the mitral isthmus in patients with persistent atrial fibrillation. Radiofrequency energy was delivered at a maximum power output of 25 - 35 W and target temperature of 55 ºC. At the target sites RF energy was applied until maximum local electrogram amplitude decreased by >50% or to <0.1 mV. PV isolation was confirmed by using a LASSO catheter positioned inside the isolated PV.

### Measurement of plasma BNP

BNP level were obtained from the antecubital bein in the supine position one day before scheduled PVI. BNP level was determined using a chemi-luminescent immunoassay for human BNP (Roche Diagnostics, Mannheim, Germany). The reference value of plasma BNP was <100 pg/dl.

### Statistical analysis

All data analysis was performed using the SigmaStat for Windows version 3.0 software. Data are expressed as medians, comparisons of the continous variables between the groups were analyzed using Student's t-test or ANOVA, as appropriate. Statistical significance was selected as a value of p<0.05.

## Results

### Pulmonary vein isolation and follow up

Successful isolation of all pumonary veins was performed in all 68 patients (48 patients with paroxysmal AF, 20 patients with persistent AF). After three months all patients (100%) received a 7-days-holter monitoring. 38 (79,1%) patients with paroxysmal AF had stable sinus rhythm without any antiarrhythmic medication, while in 10 patients (20,9%) a recurrence of AF or the appearence of a left atrial tachycardia due to the ablation could be observed. In the group of patients with persistent AF 11 (55%) patients showed no recurrence of AF while in 9 patients (45%) paroxysmal or persistent AF could be detected.

### BNP  plasma level

**Figure 1:**
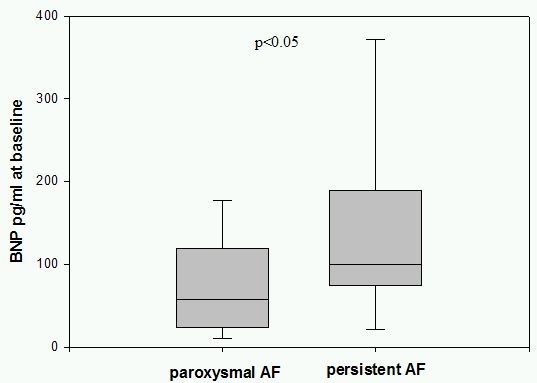
BNP in patients with paroxysmal and persistent AF. BNP plasma levels before PVI were elevated in patients with persistent AF compared to patients with paroxysmal AF significantly.

**Figure 2:**
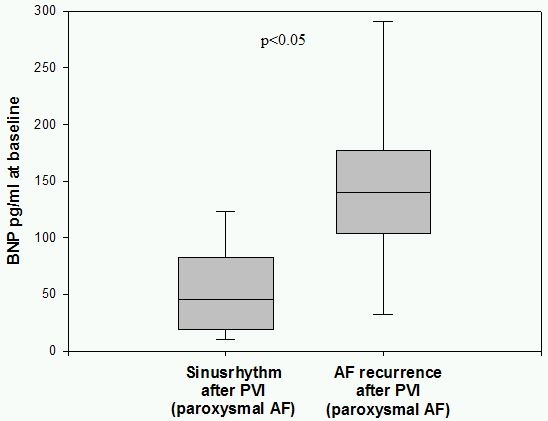
BNP in paroxysmal AF patients grouped by PVI success. Patients with paroxysmal AF and stable sinusrhythm after PVI  showed significant lower BNP levels at baseline compared to patients with recurrent AF.

**Figure 3:**
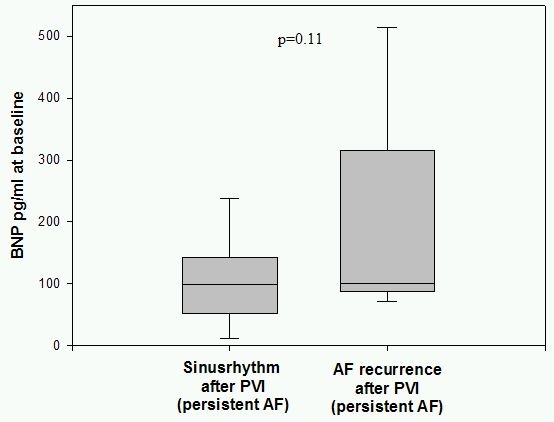
BNP in persistent AF patients grouped by PVI success. In patients with persistent AF and successful PVI plasma levels of BNP at basline were lower than in patients with recurrent AF.

BNP plasma levels in patients with paroxysmal AF were normal at baseline with 84,4 pg/ml. In contrast in patients with persistent AF BNP at baseline was significanly higher with 145,5 pg/ml (p<0.05) and is shown in Figure 1. Patients with paroxysmal AF and recurrence of AF after PVI showed significantly higher BNP plasma levels at baseline (144,1 pg/ml) compared to 68,7 pg/ml (p<0.05) in patients with stable sinus rhythm as shown in Figure 2.  In the group of patients with persistent AF successfully treated patients had lower BNP levels with 105,8 pg/ml at baseline versus 193,3 pg/ml in patients with recurrent AF (p=0.11) as shown in Figure 3. Patients characteristics concerning age, duration of paroxysmal / persistent AF and left atrial volume showed no significant differences in both groups of patients with normal baseline BNP levels <100pg/ml or elevated levels >100pg/ml as shown in Table 1.

## Discussion

Cardiac natriuretic peptides like BNP play an important role in the regulation of volume hemostasis. Studied mainly as a marker of congestive heart failure in structural heart disease [3,4] its diagnostic potential in patients with recurrent arrhythmias like AF remains unclear. Several studies have shown that even in patients with normal left ventricular ejection fraction and without clinical signs of congestive heart failure, BNP levels can be elevated due to an atrial overload and atrial remodelling caused by AF [5,6]. In our study all patients with AF were free of systolic left ventricular dysfunction or structual heart disease. However in patients with persistent AF BNP levels were significantly higher probably due to a higher grade of AF burden. Treatment of AF can be very challenging since the participation of triggering factors and substrate changes of the left atrium are not fully understood. Since ablation strategies for the treatment of AF have been introduced [7] the number of patients who have been treated by ablation has raised continously, especially in patients with symptomatic paroxysmal AF. Therefore left atrial ablation therapy of AF remains a complex ablation procedure associated with both procedural risks for the patients and challenging efforts for the electrophysiologist. Regarding the major complications of stroke, pericardial tamponade or oesophagus fistula and the consumption of time and material, the effectiveness of patient selection has to be improved, especially when considering success rates of only 70%. Besides established prognostic parameters for successful restoration of sinus rhythm in patients with AF like age, atrial size or duration of AF, we have demonstrated that BNP is a  predictor of successful PV isolation in patients with paroxysmal AF. Although we could not prove the same predictive value of BNP in patients with persistent AF statistically, a tendency could be shown suggesting that BNP can also be used as predictor for successful PVI in patients with persistent AF.

## Conclusions

BNP is a predictor for successful PV isolation in patients with AF and should be determined routinely before an ablation procedure. It should be integrated in the process of patient selection for ablation of atrial fibrillation.

## Study limitations

Although all patients in this study  showed a normal systolic left ventricular function, we did not consider the possibility of a diastolic dysfunction which can contribute to an increased BNP level as well.  Due to the small number of cases included in this study the statistical analysis is limited, thus a trial with a larger cohort of patients is necessary to confirm our hypothesis, that BNP plasma levels are a feasible predictor of a succesful AF ablation procedure.

## Figures and Tables

**Table 1:**
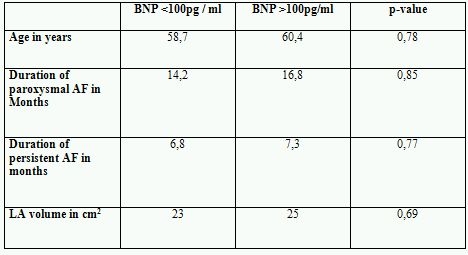
Patient characteristics in both groups with  baseline BNP plasma levels <100pg/ml and >100pg/ml showing no statistical differences.
